# Evaluating a Novel Approach to Cardiovascular Risk in Diabetes∗

**DOI:** 10.1016/j.jacadv.2024.100851

**Published:** 2024-02-17

**Authors:** Michael E. Farkouh, Parth Visrodia

**Affiliations:** a Department of Cardiology, Smidt Heart Institute, Cedars-Sinai Medical Center, Los Angeles, California, USA; b Department of Internal Medicine, Cedars-Sinai Medical Center, Los Angeles, California, USA

**Keywords:** diabetes, risk prediction

**Figure:**
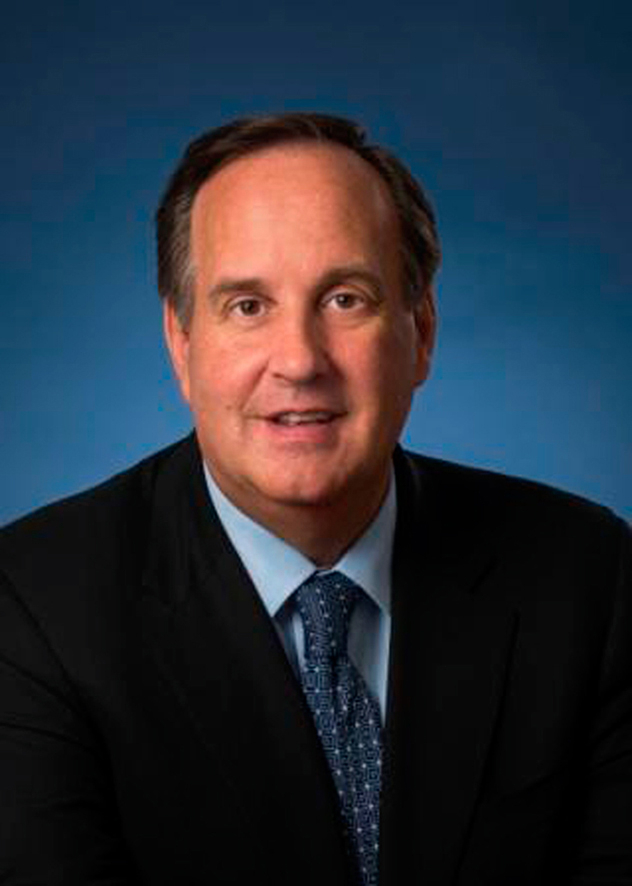

The pursuit of advancing diabetes care has brought to light the formidable challenge presented by atherosclerotic cardiovascular disease (ASCVD). As one of the leading causes of mortality and hospitalization among individuals with diabetes, ASCVD is an important complication of diabetes, which is why a proper assessment of ASCVD risk is so valuable.^1^ Currently, there are numerous risk predictors for ASCVD, but most are not customized to patients with diabetes, and many are limited in the precision of risk prediction. The most commonly used predictors are the American College of Cardiology/American Heart Association ASCVD risk calculator (also known as the pooled cohort equation) and the Framingham Risk Score.^2^, ^3^, ^4^, ^5^ Guidelines for therapies have been adopting these risk models, but they have not received adequate uptake in clinical practice due to complexity in ascertainment and lack of uniform adoption of electronic medical record platforms.^6^

In this issue of *JACC: Advances*, McCoy et al.^7^ propose a new risk prediction model, the Annualized Claims-Based MACE Estimator (ACME), as a more accurate way to predict ASCVD risk than traditional risk calculators. The authors highlight important deficiencies and limitations in current ASCVD risk calculators, including the pooled cohort equation. Many of them were not developed specifically for patients with diabetes, were designed to estimate only 10-year risk rather than shorter-term risk, and were limited to asymptomatic individuals without prior ASCVD. The ACME addresses many of these deficiencies.

The study cohort uses retrospective data from OptumLabs Data Warehouse linked to a 100% sample of Medicare fee-for-service to focus exclusively on adults with type 2 diabetes and includes a sample size of over 6 million with a diverse mix of ages, racial and ethnic groups, income levels, geographic regions, and different health plans that supports its generalizability. The study uses claims data, which helps avoid biases associated with cohort enrollment and prospective participation. The study cohort also includes patients with type 2 diabetes with a spectrum of risk for cardiovascular disease (CVD), including those with moderate risk of CVD. Since many trials of CVD risk reduction focus on patients at either low or high risk for CVD, this study can help address the gaps in those with moderate risk for CVD, which is the majority of patients with type 2 diabetes. This, along with the model’s ease of implementation, helps set the model to be integrated in future clinical trials so that cardiovascular risk reduction can be better assessed. The model also offers more flexibility in the time span of ASCVD risk assessment, as it can assess risk over much shorter time intervals rather than a fixed 10-year span.

Ultimately, the ACME risk model does appear to be superior to current ASCVD risk calculators in certain aspects, though it is difficult to directly compare them as they were all developed in different patient populations. McCoy et al.^7^ found that 22 of the most commonly used ASCVD risk models have a Harell’s concordance index below the concordance of the ACME model, suggesting that the ACME has greater predictive accuracy in discriminating between individuals with different risks of experiencing a major adverse cardiovascular event.

McCoy et al.^7^ make note of important limitations in the study, including its generalizability and lack of use of some clinical data. Since the study uses retrospective data to focus on patients with type 2 diabetes in the United States, its external validation to patients without health insurance (which may be more than 10% of patients with diabetes)^8^, other countries, and different clinical complexities is limited. However, perhaps the most significant limitation is that the study does not use certain clinical data, including blood pressure, lipid levels, and glycemic control, all of which have been shown to have a strong link to ASCVD risk and have been included in other risk predictors.^3^^,^^9^ Not having this data leaves a potential gap in the ACME model, where it may assign the same ASCVD risk to 2 patients despite having completely different lipid levels and blood pressures. Other limitations include temporal changes in diabetes management. The study uses retrospective data spanning from 2014 to 2021, a time during which many novel medications have evolved or gained popularity, including sodium-glucose cotransporter 2 inhibitors and glucagon-like peptide-1 agonists, both of which have demonstrated cardiovascular risk reduction.^10^^,^^11^ However, the dataset used by this model is still more current than other risk assessment models. In addition, the 7-year time frame also limits the ability of the model to assess ASCVD risk over a more extended time period compared to other models, like the pooled cohort equation.

This model introduces a novel way to assess ASCVD risk in patients with type 2 diabetes and can be improved and adopted widely with some additions. Future iterations of ACME should focus on adding clinical data as well as other types of nonclinical data to provide a more holistic view of ASCVD risk assessment. Important clinical data to include would be blood pressure, lipid levels, and glycemic control, given how strongly they are associated with ASCVD risk. Nonclinical data that could be incorporated that impacts ASCVD risk assessment would include socioeconomic determinants of health. Other nonclinical data to add would include some patient-reported outcomes, which, although not as objective and susceptible to reporting biases, can help further individualize a patient’s ASCVD risk. Lifestyle factors, such as patients’ exercise, diet, and medication adherence can have a significant impact on ASCVD risk, and incorporating them into a risk model could encourage physicians to discuss them more with patients.

Overall, the ACME addresses a current gap in assessing ASCVD risk in patients with type 2 diabetes and has significant strengths that are lacking in current models. While it has its limitations, the most significant of which is the lack of clinical data, it serves as an excellent foundation that can be built upon and incorporated into future clinical trials and care pathways to help advance diabetes care.

## Funding support and author disclosures

The authors have reported that they have no relationships relevant to the contents of this paper to disclose.
